# Managing the Personal Side of Health: How Patient Expertise Differs from the Expertise of Clinicians

**DOI:** 10.2196/jmir.1728

**Published:** 2011-08-16

**Authors:** Andrea Hartzler, Wanda Pratt

**Affiliations:** ^1^Division of Biomedical and Health InformaticsSchool of MedicineUniversity of WashingtonSeattle, WAUnited States; ^2^The Information SchoolUniversity of WashingtonSeattle, WAUnited States

**Keywords:** Health knowledge, attitudes, practice, social support, community networks, peer group, consumer health informatics, online communities, patient expertise, personalized health

## Abstract

**Background:**

When patients need health information to manage their personal health, they turn to both health professionals and other patients. Yet, we know little about how the information exchanged among patients (ie, patient expertise) contrasts with the information offered by health professionals (ie, clinician expertise). Understanding how patients’ experiential expertise contrasts with the medical expertise of health professionals is necessary to inform the design of peer-support tools that meet patients’ needs, particularly with the growing prevalence of largely unguided advice sharing through Internet-based social software.

**Objective:**

The objective of our study was to enhance our understanding of patient expertise and to inform the design of peer-support tools. We compared the characteristics of patient expertise with that of clinician expertise for breast cancer.

**Methods:**

Through a comparative content analysis of topics discussed and recommendations offered in Internet message boards and books, we contrasted the topic, form, and style of expertise shared in sources of patient expertise with sources of clinician expertise.

**Results:**

Patient expertise focused on strategies for coping with day-to-day personal health issues gained through trial and error of the lived experience; thus, it was predominately personal in topic. It offered a wealth of actionable advice that was frequently expressed through the narrative style of personal stories about managing responsibilities and activities associated with family, friends, work, and the home during illness. In contrast, clinician expertise was carried through a prescriptive style and focused on explicit facts and opinions that tied closely to the health care delivery system, biomedical research, and health professionals’ work. These differences were significant between sources of patient expertise and sources of clinician expertise in topic (*P* < .001), form (*P* < .001), and style (*P* < .001).

**Conclusion:**

Patients offer other patients substantial expertise that differs significantly from the expertise offered by health professionals. Our findings suggest that experienced patients do not necessarily serve as “amateur doctors” who offer more accessible but less comprehensive or detailed medical information. Rather, they offer valuable personal information that clinicians cannot necessarily provide. The characteristics of patient expertise and the resulting design implications that we identified will help informaticians enhance the design of peer-support tools that will help meet patients’ diverse information needs.

## Introduction

In addition to the indispensable information received from health professionals, patients use information and advice offered by other patients to help them actively participate in their own health care and make informed personal health decisions. Although patients are best known for providing emotional support, they also offer other patients personal health guidance based on the expertise they have gained from managing similar health situations. We define *patient expertise* as experiential knowledge gained from personally managing the day-to-day experience of illness. For example, patients develop expertise in the self-management of chronic conditions through their everyday experience with self-care over time [1-3]. Some experienced patients can even come to know as much as their doctors about aspects of their health [4]. In the context of breast cancer, patient expertise reflects practical know-how and coping strategies exchanged among patients and their peers, including other patients, cancer survivors, and their caregivers, family, and friends (ie, other patients). In contrast, we define *clinician expertise* as knowledge gained from professional training and practice. Clinician expertise is shared with patients by health professionals (eg, physicians, nurses, therapists, and support staff).

In contrast to other forms of social support, including *emotional* support (ie, communication of caring and concern) and *instrumental support* (ie, provision of material goods), patients commonly share their expertise through *informational support,* which involves the provision of information and advice used to guide one’s personal health management in new ways [5]. A patient’s need for informational support is thought to be strongest during periods of transition, once emotional support needs associated with a crisis have been met [6]. The need for guidance, which is carried through informational support, is commonly met by people with expertise [7]. In the context of patient-expertise sharing, experienced patients serve as experts by providing informational support for personal health management through experiential guidance. Peer-support programs for cancer patients that provide opportunities to exchange such guidance are associated with benefits for patients, including improved coping skills, understanding of the cancer experience, and psychosocial outcomes [8,9].

Patient expertise has been valued in varied and growing contexts. For example, personal knowledge, such as lifestyle, priorities, and experiences, is an important contribution patients make to shared decision making with health professionals [10]. Providing patients with decision-support tools to share their personal knowledge and experiences with health professionals can improve nursing care and patient outcomes [11]. Other research demonstrates the value of involving patients as teachers who share their illness experiences through medical education [12].

In this work, we focus on patients sharing their expertise with one another. Breast cancer patients, for example, have expressed a strong need for experiential health information provided by peers [13]. When those needs for patient expertise are met, patients might be better able to receive and appropriately use health information than when patients interact with an “ask-the-expert” service that offers clinically oriented resources [14]. However, we lack a deep understanding of the characteristics of patient expertise, which hinders clear guidance on how to design peer-support tools that facilitate patient-expertise sharing.

Historically, patients who share similar health situations have helped one another to cope with illness by sharing their expertise through participation in patient-led support groups [15], as mentors in pioneering programs such as “Reach to Recovery” [16], and as instructors for chronic disease self-management programs [17]. Although the Internet has facilitated expertise sharing among patients in online health communities, many content analyses of interactions among correspondents of online health communities for conditions such as breast cancer [18-22] focus on emotional support [23]. Yet, growing evidence highlights the high prevalence of patient expertise exchanged through informational support in online health communities [24-26].

Patient expertise has continued to gain visibility as Web-based social software (eg, forums, social networking tools, blogs, and wikis) helps patients to readily exchange information and advice with others who are facing similar health situations [27-31]. For example, patient-led support groups provide a longstanding online resource for patient-generated guidance and advice on treatments, personal histories, shared experiences, and medication side effects for epilepsy [24]. More recently, personal profiles and commenting features of PatientsLikeMe.com allow users to ask one another about specific health experiences and to offer advice, suggestions, and tips that stem from those experiences [32]. In a recent survey, 1 in 5 Internet users reported going online to find others with similar health concerns, particularly for chronic or rare conditions [31]. Indeed, many patients now use social software more often to obtain expertise from other patients than to obtain their emotional support [28]. However, social software still needs enhancements to facilitate this important peer interaction around health [33], particularly in making the expertise of patients more prominent, explicit, and accessible [34]. For example, tools could do more to help users relate to the health experiences of other users [35] or to help users gain awareness of the expertise available from other users without the time required for multiple interactions when building relationships [9]. Research to improve our understanding of the characteristics of patient expertise could deeply inform design enhancements that facilitate expertise sharing.

Despite the growing prevalence of expertise sharing among patients on the Internet, disparate views about the characteristics of that expertise remain. For example, Meier et al [25], through a content analysis of cancer-related Internet mailing lists, found that patient exchanges of information and advice clustered squarely around medically oriented topics (eg, treatments and communication with health care providers). Our preliminary work on breast cancer message boards shows additional clusters around personal topics related to the cancer experience, including the family, home, work, advocacy, and interactions with social networks [26]. Similarly, other work notes that nearly half of interactions between cancer patients and survivors through a telephone-based peer-support system revolved around psychosocial and day-to-day issues, such as the impact of cancer on family and friends, compared with interactions revolving around more medically focused treatments and side effects [36]. A mixture of treatment-related and personal topics has also been noted in discussions among other patient groups, such as questions posed to peers on online forums for epilepsy [24], amyotrophic lateral sclerosis [37], and anterior cruciate ligament injuries [23]. These contrasting views of patient expertise (ie, whether it is more medial or more personal in nature) warrant detailed investigation that explores how the expertise exchanged among patients contrasts with the medical expertise offered by health professionals.

Facilitating patient-expertise sharing with innovative technology will depend on a solid understanding of the fundamental characteristics of the expertise that patients share. For example, could we meet patients’ needs for information solely by enhancing communication between patients and health professionals? Alternatively, do patients also need help finding other patients who have had similar health experiences because clinicians have neither the time nor the expertise to meet all their needs? An important first step to answering these questions is to understand the role of both patient expertise and clinician expertise in meeting patients’ needs.

Our aim in this study was to enhance our understanding of patient expertise by assessing how it differs from clinician expertise. In the context of breast cancer, we conducted an in-depth and comparative content analysis [38] to investigate how patient expertise and clinician expertise compare with respect to topics discussed and the types of recommendations offered. Through a multiphased approach, we enhanced our previous work [26] by (1) extending our characterization of patient expertise through the analysis of content from 2 patient-authored books, (2) characterizing clinician expertise through the analysis of content from a leading breast cancer resource book written by a health professional and an “ask-the-doctor” message board, and (3) contrasting the characteristics of patient expertise and clinician expertise. We conclude with suggestions for how our results can be used to enhance the design of collaborative peer-support tools for patients that will help them cope with their health issues and make informed health care decisions.

## Methods

Using an evolving coding scheme that was grounded in the data [39], we conducted our content analysis of patient and clinician expertise in 4 phases. In phase 1, we analyzed content from sources of patient expertise to identify emergent *topics* discussed (ie, medical or personal) and the types of *recommendations* offered (ie, advice in the form of suggested action strategies, knowledge, perspectives, or information resources). We also noted the style through which recommendations were carried (ie, narrative or prescriptive). Next, in phase 2, we expanded our coding scheme by repeating this procedure using content from sources of clinician expertise. We then solidified our coding scheme into a codebook and tested the reliability of our coding procedure in phase 3. Finally, in phase 4, we contrasted the topics discussed and recommendations offered in sources of patient expertise and sources of clinician expertise. We describe each phase in detail in the Procedure section. This multiphased analysis answered 2 main research questions: (1) How do topics discussed in sources of patient expertise compare with topics discussed in sources of clinician expertise? (2) How do the recommendations offered by sources of patient expertise compare in form and style with recommendations offered by sources of clinician expertise?

### Content Sources

We analyzed sources of patient expertise and sources of clinician expertise from both online message boards and books. Message boards are common online resources for patients to seek advice from peers through online communities or to seek advice from health professionals through ask-the-expert forums. Books are traditional resources that patients also commonly turn to for advice, both authored by health professionals (ie, clinician expertise) and authored by peer cancer survivors (ie, patient expertise). Books are particularly important because they are one of the few written forms available to patients who have no Internet access. Although books offer a limited source of perspectives because of the short list of authors, they attempt to provide in-depth expertise from that perspective. In contrast, message boards bring insights into the kinds of expertise actively sought by patients from a breadth of perspectives. Although patients have available to them a spectrum of valuable resources, our combined analysis of message boards and books enabled us to capture a variety of expertise that is available to and sought by patients at both ends of that spectrum, both online and offline.

Sources of patient expertise in our analysis included 3 online message boards that support correspondence among breast cancer patients, and 2 books written by cancer survivors. We selected the 3 patient message boards (message boards A, B, and C) based on public accessibility, high volume of use, longevity, and variation in formality (ie, varied levels of moderation and affiliation with health-related organizations). We selected the 2 patient books because they are autobiographical yet differ in style. The first patient book (book 1) is highly narrative in its compilation of experiences contributed by 10 breast cancer survivors. The second patient book (book 2) is an interactive guide written by a 6-year survivor of metastatic cancer who provides extensive strategies for staying organized and informed during the cancer experience.

Sources of clinician expertise included an ask-the-doctor message board that supports correspondence between breast cancer patients and health professionals. We selected this message board over clinical advice summaries or health professionals’ blogs to enable analysis of questions from patients and answers from health professionals. As an additional source of clinician expertise, we chose *Dr. Susan Love’s Breast Book* [40], which is a popular book written for breast cancer patients. We selected this book because correspondents in the message boards we analyzed often recommended this popular resource to one another. For example, this book was referred to as the “bible” of breast cancer and is a source of clinician expertise that many patients turn to.

Table 1 shows the content sources, including the number of text pages we analyzed and the number of content units (see Procedure) that each source contributed to the analysis. Table 2 shows characteristics of the 4 message boards.

**Table 1:** Content sources

	Source	Text pages	Content units
Patient expertise	Message board A	174	50
	Message board B	316	50
	Message board C	276	50
	Book 1: McCarthy and Loren, 1997 [41]	230	79
	Book 2: Willis, 2001 [42]	220	131
	Total	1216	360
Clinician expertise	Ask-the-doctor message board	277	150
	Book: Love and Lindsey, 2000 [40]	552	225
	Total	829	375

**Table 2:** Characteristics of message boards

	Message board			
	A	B	C	Ask the doctor
Year of inception	1998	1994	1998	2000
Moderation	Yes	No	No	Yes
Affiliation with a health-related organization	Yes	Yes	No	Yes
Total messages	379	152	316	300
Mean messages/thread (range)	8 (1–31)	3 (1–10)	8 (1–27)	2 (1–2)
Days’ worth of threads	5	24	55	85

### Procedure

In phase 1, we analyzed content from the sources of patient expertise. Our unit of analysis for message boards was the *thread* (ie, 1 or more related messages) and for books was the *subsection* (ie, a titled section within a chapter). Our inclusion criteria for the analysis were *content units* (ie, message board thread or book subsection) that solicited or offered informational support (ie, information used to guide or advise) related to the diagnosis, treatment, or long-term management of breast cancer.

We collected archived threads from the patient message boards with posting dates starting in February 2006 until we obtained 50 content units from each board that met our inclusion criteria. Obtaining an equal number of content units from each patient message board required the collection and filtering of more threads from message board B (130 threads) than from message board A (66 threads) or message board C (81 threads). Common kinds of threads that we excluded from the analysis reflected exchanges of pure emotional support, technical support issues, threads labeled by correspondents as *off topic*, and spam-like advertisements. For our corresponding examination of patient expertise in books, we divided both patient books into subsections. All subsections from both patient books met our inclusion criteria. Sources of patient expertise contributed 360 content units in total. The patient message boards contributed 150 content units and patient books contributed 210 content units (see Table 1).

Based on themes that emerged from our preliminary analysis of informational support exchanged in the patient message boards [26], we coded content units from the sources of patient expertise while expanding our coding scheme. Our coding procedure was shaped by the challenge of identifying explicitly formulated questions within long, evolving discussion threads on patient message boards. In our preliminary work, we noticed that questions were often formulated as threads evolved. Other times, discussion was initiated by comments, rather than questions. More generally, we observed that threads typically discussed an overarching problem, or “topic,” whether initiated by an explicit question, an evolving question, or a comment. In response, other users would post potential solutions, or “recommendations,” for that problem. Rather than limiting our analysis to only those threads initiated by explicitly formulated questions, we framed our coding procedure more generally in terms of topics and recommendations that content units reflect. Thus, for each content unit, we used our evolving coding scheme to capture emergent topics discussed and recommendations offered:

- **Topics** reflect a spectrum of personal health issues discussed, such as specific situations or problems (eg, choosing a doctor). Topics represent the predominate theme of a content unit, which was typically most clearly identified within the introductory paragraph of a book subsection or within the initiating message and subject line of a message board thread. We assigned 1 topic to each content unit.
- **Recommendations** reflect a range of advice offered for dealing with the personal health issue (ie, topic) discussed within a content unit, such as a suggested solution to a problem. Unlike the breadth captured by topics, recommendations are fine-grained statements of advice serving as potential problem solutions in the form of action strategies, knowledge, perspectives, and information resources that were often sprinkled throughout a content unit. Recommendations reflect either a prescriptive style (ie, “you should...”) or a narrative style when carried through a personal story (ie, “when I was in that situation, this is what I did...”). We assigned 1 or more recommendation to each content unit.

In **phase 2**, we expanded the coding scheme by repeating our coding procedure on content from sources of clinician expertise. We collected threads from the ask-the-doctor message board until we obtained 150 content units that met our inclusion criteria. Unlike the threads from the patient message boards, the threads from the ask-the-doctor message board were generally short, consisting of a question posted by a patient and a response posted by a health professional, and each met our inclusion criteria. We divided the clinician book into subsections and excluded subsections that did not meet our inclusion criteria. We excluded subsections from chapters 1 through 9 of the clinician book because content from those chapters describes the development of healthy breasts and common breast problems rather than relating directly to the diagnosis, treatment, or long-term management of breast cancer. Sources of clinician expertise contributed 375 content units in total. The ask-the-doctor message board contributed 150 content units and the clinician book contributed 225 content units (see Table 1).

In **phase 3**, we used card sorting [43] and discussion to solidify our evolving coding scheme into a codebook made up of 2 main overlapping topics (medical and personal) and 4 types of recommendations (action strategies, knowledge, perspectives, and information resources). Our codebook, which reflects the end point of our evolving coding scheme, includes clusters of subtopics discussed, as well as different styles (ie, prescriptive or narrative) through which recommendations were expressed across all content units. Table 3 shows our codebook and details the distribution of topics (part a) and recommendations (part b) in patient and clinician sources. We describe the codes making up our codebook in greater detail in the Results section.

**Table 3:** Codebook of (a) topics and (b) recommendations (percentages reflect proportions of content units from each type of content source)

			Patient message boards	Patient books	Ask the doctor	Clinician book
**a. Topics**						
	**Medical**					
		Deciding on care teams, treatments, and procedures	16 (11%)	9 (4%)	19 (13%)	14 (6%)
		Understanding biomedical concepts and processes	49 (33%)	6 (3%)	102 (68%)	145 (65%)
		Managing interactions with health professionals	2 (1%)	15 (7%)	17 (11%)	1 (0.5%)
		Managing information to collaborate with clinicians or understand biomedical issues	2 (1%)	3 (1%)	6 (4%)	5 (2%)
	**Personal**					
		Managing life at home	8 (5%)	16 (8%)	0 (0%)	0 (0%)
		Managing work life	3 (2%)	14 (7%)	1 (0.7%)	1 (0.5%)
		Managing one’s emotional response to illness	12 (8%)	11 (5%)	0 (0%)	5 (2%)
		Managing interactions with social networks	8 (5%)	18 (9%)	1 (0.7%)	3 (1%)
		Managing personal tasks and projects	16 (11%)	86 (41%)	1 (0.7%)	19 (9%)
		Managing advocacy and volunteer work	6 (4%)	2 (1%)	0 (0%)	0 (0%)
	**Both medical and personal**		28 (19%)	30 (14%)	3 (2%)	32 (14%)
		Total content units	150	210	150	225
**b. Recommendations**							
	**Action strategies**						
		Prescriptive	248 (23%)	303 (14%)	122 (35%)	474 (13%)	
		Narrative	192 (18%)	223 (10%)	0 (0%)	27 (1%)	
	**Knowledge**						
		Prescriptive	159 (15%)	419 (19%)	225 (65%)	1,620 (45%)	
		Narrative	204 (19%)	264 (12%)	0 (0%)	133 (4%)	
	**Perspectives**						
		Prescriptive	96 (9%)	70 (3%)	0 (0%)	76 (2%)	
		Narrative	48 (4%)	97 (4%)	0 (0%)	3 (<1%)	
	**Information resources**						
		Books	13 (1%)	11 (1%)	0 (0%)	195 (6%)	
		Contact information	17 (2%)	23 (1%)	0 (0%)	314 (9%)	
		Magazines and magazine articles	2 (<1%)	7 (<1%)	0 (0%)	15 (<1%)	
		Multimedia	0 (0%)	4 (<1%)	0 (0%)	140 (4%)	
		News articles	19 (2%)	11 (1%)	0 (0%)	2 (<1%)	
		Poems and quotes	5 (<1%)	24 (1%)	0 (0%)	0 (0%)	
		Research articles and academic journals	11 (1%)	64 (3%)	0 (0%)	350 (10%)	
		Templates	0 (0%)	115 (5%)	0 (0%)	4 (<1%)	
		Webpages	70 (6%)	482 (22%)	1 (<1%)	118 (3%)	
		Miscellaneous publications	0 (0%)	86 (4%)	1 (<1%)	102 (3%)	
		Total recommendations	1084	2203	349	3573	

We used the codebook to test the reliability of our coding procedure using a 10% reliability sample of content units. Based on the number of contributing units, we randomly selected a set of content units from each content source for the reliability sample. An independent coder (CL) applied the codebook to the reliability sample. We calculated kappa scores to determine the level of intercoder agreement between codes applied to the reliability sample by AH (1 of the authors) and by CL. We applied linear weighting to our kappa calculations [44] for recommendations to account for the level of agreement between coders for both types and numbers of recommendations (ie, coders could assign multiple types and numbers of recommendations to each content unit). Reliability test results show good intercoder agreement for topics (κ = .71), action strategies (κ = .69), knowledge (κ = .72), and perspectives (κ = .54), as well as excellent intercoder agreement for information resources (κ = .94).

In phase 4, we compared the kinds of topics discussed and the types of recommendations offered in sources of patient expertise versus sources of clinician expertise. We compared the distribution of topics and recommendations across patient sources and across clinician sources. Then, we explored differences in the proportions of subtopics as well as the types and styles of recommendations among content sources. Finally, we used Pearson’s chi square statistic to assess differences in the frequencies of topics and recommendations between sources of patient expertise and sources of clinician expertise.

### Ethical Considerations

We thought deeply about ethical considerations and evolving guidelines for conducting Internet-based research [45-49] as we obtained, analyzed, and reported our findings from online message board content. Thus, we selected public message boards for which membership was not required to access content, collected archived threads, removed identifiers from collected threads, and took care in reporting our results to balance the anonymity of correspondents with research trustworthiness. Our approach aligns closely with the approach taken in other content analyses of online health message boards, such as Finn [50]. We obtained institutional review board approval through the University of Washington before collecting data from the message boards.

## Results

We analyzed 735 content units across all sources. Patient sources contributed 360 content units and clinician sources contributed 375 content units. Each content unit was associated with 1 topic, but usually with many recommendations. Content units contained 7209 recommendations in total. Content units from patient sources contained 3287 recommendations and content units from clinician sources contained 3922 recommendations. Table 4 shows the distribution of topics (part a) and recommendations (part b) across individual content sources.

**Table 4:** Distribution of (a) topics and (b) recommendations across content sources (percentages reflect proportions from individual sources)

		Patient message boards			Patient books		Ask the doctor	Clinician book
		A	B	C	1	2		
**a. Topics**								
	Medical	25 (50%)	12 (24%)	32 (64%)	12 (15%)	21 (16%)	144 (96%)	165 (74%)
	Personal	18 (36%)	22 (44%)	13 (26%)	58 (73%)	89 (68%)	3 (2%)	28 (12%)
	Both	7 (14%)	16 (32%)	5 (10%)	9 (12%)	21 (16%)	3 (2%)	32 (14%)
	Total content units	50	50	50	79	131	150	225
**b. Recommendations**								
	Action strategies	215 (39%)	119 (47%)	106 (38%)	300 (36%)	226 (17%)	122 (35%)	501 (14%)
	Knowledge	200 (36%)	52 (21%)	111 (40%)	368 (44%)	315 (23%)	225 (64%)	1753 (49%)
	Perspectives	86 (15%)	33 (13%)	25 (9%)	121 (14%)	46 (3%)	0 (0%)	79 (2%)
	Information resources	54 (10%)	47 (19%)	36 (13%)	49 (6%)	778 (57%)	2 (1%)	1240 (35%)
	Total recommendations	555	251	278	838	1365	349	3573

Next, we detail the kinds of topics and recommendations that emerged from our analysis across content units from all sources. The descriptive detail we provide on topics and recommendations corresponds to the codes making up our codebook (see Table 3). We then describe how sources of patient expertise and sources of clinician expertise compare with respect to those topics and recommendations.

### Topics Discussed Across Content Sources

Most content units fell into 2 broad topic categories: discussion that was mostly *medical* in nature (411/735 content units, 56%) or discussion that was mostly *personal* in nature (231/735 content units, 31%). A smaller proportion of content units contained discussion that shared aspects that were *both medical and personal* in nature (93/735 content units, 13%). Next, we provide representative quotes to describe the kinds of personal health issues that emerged as subtopics in each topic category.

#### Medical Topics

Topics that were medical in nature involved problems or concerns about constructs or processes that are tied closely to the health care delivery system, biomedical research, and health professionals’ work. Medical topics often reflected discussion that could stimulate an improved understanding of the medical domain or strategies to better fit one’s life to the health care delivery system. Common clusters of subtopics that fell in the medical category with representative examples include the following:

(a) Deciding on health care teams, treatments and procedures, and research trial enrollment

- “Tackling the selection of our health care team”
- Being in a “dilemma about reconstruction”
- Dealing with competing recommendations from different doctors
- Deciding between “radiation and tamoxifen or chemo and radiation”
- Deciding whether to have a biopsy
- Determining eligibility for participation in “genetic research*.* *”*

(b) Understanding biomedical concepts and processes, clinical treatments, procedures and tests, side effects, and biomedical research

- Understanding “cancer staging” and other medical terminology
- Determining whether a “bone scan” is a typical part of cancer care
- Discussing a “pathology report question”
- Uncovering how the diagnostic process typically flows
- Understanding effects of Arimidex on cholesterol.

(c) Managing interactions with health care professionals

- “Good care is also about communication”
- Determining when to seek a second opinion
- “What can I expect” for my upcoming appointment
- Understanding considerations doctors make when recommending treatments.

(d) Managing information to collaborate with clinicians or to understand biomedical issues

- Tracking medications, pain, or side effects to share with your health care provider
- *“*Questions to ask your oncologist”
- Preparing information for appointments
- “I was supposed to take the reports to a general surgeon, but I wonder if this is necessary, since nothing was found?”
- Discussing a research article on the effectiveness of Herceptin.

#### Personal Topics

Topics that were personal in nature involved problems or concerns around constructs or processes that are tied closely to one’s personal life, including ongoing responsibilities and day-to-day activities associated with family, friends, the home, work, and health-related activities that occur outside of the health care delivery system. Personal topics often reflected discussion that could stimulate the development of practical strategies to fit health management into one’s ongoing life. Common clusters of subtopics that fell in the personal category with representative examples include the following:

(a) Managing life at home

- Recovering from medical treatments and procedures: “What to expect following surgery”
- Keeping up with family and household responsibilities: Sharing my experiences with hospice planning
- Maintaining oversight of legal, financial, and insurance issues: how to “keep track of your medical expenses.”

(b) Managing life at work

- Shifting your work load during treatment: “Worry about health, not your job performance”
- Considering the impact of cancer on work prospects and insurance: *“*We are uncertain about what would happen if we were to change employers or careers”
- Interacting with coworkers, colleagues, or clients during treatment: “Maintaining a work persona”
- Deciding whether to work during treatment: “Have any of you gone back to work during part of your chemo?”

(c) Managing the emotional response to cancer

- Coping with anxiety, anger, depression, and fear
- “Finding ways to cope with the emotional roller coaster”
- Managing the “fear of recurrence”
- “Humor is a necessary healing component.”

(d) Managing interactions with one’s social network

- “What to tell your children”
- The “Fears of our loved ones”
- Getting help to find others with a similar diagnosis
- “Letting our partners know what we expected and needed.”

(e) Managing personal tasks and projects

- Managing lifestyle and self-care, including diet, exercise, and meditation: the “Dixie cup method” to organize medications and supplements; dealing with scalp pain while losing one’s hair; seeking a good “self-massage video”
- Focusing on spirituality and hobbies
- Managing personal health information: “Identifying and utilizing information resources.”

(f) Managing advocacy and volunteer work

- Sharing notices about upcoming cancer-related fundraisers
- Reaching out to others: “Breast cancer has helped us discover our mission and taught us that we can make a difference.”

#### Both Medical and Personal

We placed content units that shared medical and personal topics fairly equally into the overlapping category *both medical and personal*. Some examples that fell in this category include the following:

(a) Understanding biological concepts and processes AND Managing interactions with one’s social network

The risk of developing breast cancer is higher for women who have family history of cancer...Telling our mothers about our diagnosis and anticipating their responses were a source of major concern and anxiety for all of us.

(b) Managing interactions with health care professionals AND Managing personal tasks and projects

After all of your treatments are completed...write down how you feel in general terms about once a month. Not only will it assist you in communicating with your doctor but it will also give you a barometer by which to measure your progress.

(c) Deciding on treatments and procedures AND Managing work life

Schedule your chemotherapy right before the weekend so that it interferes with work as little as possible.

### Recommendations Offered Across Content Sources

Recommendations offered across content units fell into 4 major types: action strategies (1589/7209 recommendations, 22%), knowledge (3024/7209 recommendations, 42%), perspectives (390/7209 recommendations, 5%), and information resources (2206/7209 recommendations, 31%). Whereas recommendations in the form of action strategies offered procedural knowledge through suggested tasks (ie, “things to do”), recommendations in the form of knowledge offered declarative knowledge through facts and opinions (ie, “things to know”). Perspectives recommended attitudes or belief systems (ie, ways of believing or approaching situations), and information resources recommended tangible artifacts (ie, “things to obtain and use”). We describe each type of recommendation in greater detail below.

During our analysis we also recognized style differences between the recommendations; some action strategies, knowledge, and perspectives were direct, or *prescriptive*, in nature (ie, “you should...”), while others were carried implicitly through the *narrative* style of personal stories (ie, “when I was in your situation, I...”). We also recognized occasional overlap between action strategies, recommended knowledge, and perspectives. For example, taking action (ie, action strategy) can rely on acquiring knowledge. We can also learn (ie, knowledge) through our actions. Although chunks of text in a content unit could contain combinations of related recommendations in these different forms, we broke the text down (eg, sentence level) to code each recommendation with the type it best fit rather than allowing overlap between these types of recommendations.

#### Action Strategies: Things to Do

Action strategies are recommended tasks to deal with a personal health issue. This procedural knowledge about specific and actionable tasks can contribute toward solving a problem*—*for example, “It may be helpful for her to meet with a radiation oncologist before the surgery to discuss the pros and cons [of mastectomy versus lumpectomy].” Prescriptive action strategies reflected direct instructions for prescribed actions*—*for example, “One piece of advice is to use a pillow or some sort of padding for your over the shoulder seatbelt [following mastectomy].” In contrast, narrative action strategies reflected personal stories*—*for example, “What helped me [when deciding between mastectomy and lumpectomy] was searching the Internet for photos and having various women who had been through it send me their [postsurgery] photos*.*”

#### Knowledge: Things to Know

Recommended knowledge refers to informative facts and opinions that one can learn to deal with a personal health issue. Unlike action strategies that represent tasks, recommended knowledge reflects declarative descriptions of concepts or ideas a person comes to understand*—*for example, you should know that “both lumpectomy and mastectomy require anesthesia.” Prescriptive knowledge included subjective perceptions, opinions, or prescribed facts*—*for example, “Your pathology report [describes the] tumor and...nearby lymph nodes”). We also considered descriptive explanations and term definitions as prescriptive*—*for example, “Staging breast cancer is the process of...”. Knowledge that was narrative in style included recommendations*—*for example, “The surgery for the tissue expander was painful for me.”

#### Perspectives: Ways of Believing or Approaching Situations

Perspectives are recommended belief systems, attitudes, or philosophies that drive an overarching approach for dealing with a personal health issue, such as putting one’s faith in God or acting as a strong advocate for oneself. In contrast to action strategies and recommended knowledge, perspectives reflect high-level and generalized beliefs, values, or attitudes toward an overarching experience*—*for example, “I made this [treatment] decision to be comfortable with my body.” We differentiated between perspectives that were prescriptive—for example, “I know it’s hard but I think you are actually mourning your old life...you need to give yourself time to do that”—and perspectives that were narrative in style—for example, “One of my big moments came when I really understood that everything will always be different from the ‘before’ and that I must adjust to that.”

#### Information Resources: Things to Obtain and Use

Information resources are recommendations for obtaining and using specific tools and tangible items to deal with a personal health issue. A diverse range of information resources were recommended (see Table 3), including books, contact information (eg, for health professionals, health organizations, and local services), magazines and magazine articles, multimedia (eg, videos, graphs, figures, audiotapes, calculators), news articles, poems and quotes, academic journals and research articles, templates, webpages, and miscellaneous types of publications (eg, conference papers, pamphlets, brochures, white papers, and recipes). We were struck by the diversity of recommended webpages and multimedia. Webpages ranged from cancer-related organizations to personal websites and blogs. Multimedia ranged from static figures and graphs to audio, video, and interactive programs.

Templates, which included structured lists, tables, and worksheets for correspondents to personalize by filling them in, were an unexpected type of information resource. Templates reflect an embodiment of expertise that offer scaffolding to organize thoughts or actions surrounding personal health issues, such as tracking one’s health status, side effects, medical expenditures, or day-to-day events, recording research evidence on treatments, preparing for medical procedures, or assessing one’s personal level of resilience, pain, or nutrition. In contrast to clinician-oriented templates (eg, drain logs for patients to record and communicate postsurgery recovery to clinicians) that draw upon professional expertise, templates created by patients draw upon patients’ personal health experiences. For example, patient book 2 and the clinician book both offered templates that outline considerations for choosing a clinician or care team. Both templates suggested assessing clinicians’ communication style, their involvement with clinical studies, and the ability to tape-record visits. However, the patient book also reflected the patient’s experience by recommending consideration of clinicians’ personal character, professional reputation, availability, and payment options. In contrast, the clinician book reflected the clinician’s experience by recommending consideration of clinicians’ explanations for clinical tests, their interactions around complimentary and alternative treatments, and whether they are threatened when patients bring in information from the media to discuss.

### Differences in Topics Discussed Between Sources of Expertise

Although sources of patient expertise and sources of clinician expertise contained content units that spanned both medical and personal topics, the proportions of content units falling under each topic (ie, medical, personal, or both medical and personal) differed significantly between those sources (χ^2^_2_[N = 735] = 233.4, *P* < .001). This significant difference held when we compared topics discussed in message boards alone (χ^2^_2_[N = 300] = 91.2, *P* < .001) and in books alone (χ^2^_2_[N = 435] = 168.7, *P* < .001). On average, patient sources discussed more personal topics and clinician sources discussed more medical topics. For example, the maximum proportion of content units from patient sources that discussed medical topics was 64% (32/50) on message board C, whereas the minimum for clinician sources was 74% (165/225) in the clinician book. In contrast, the minimum proportion of content units from patient sources that discussed personal topics was 26% (13/50) on message board C, whereas the maximum proportion for clinician sources was only 12% (28/225) in the clinician book (see Table 4).

Although sources of patient expertise showed a high proportion of personal topics relative to clinician sources, the degree to which personal topics were discussed varied across individual books and message boards. For example, 58 out of 79 content units (73%) from the patient book 1 contained personal topics, whereas only 13 out of 50 content units (26%) from patient message board C did so (see Table 4). In contrast, the sources of clinician expertise were both predominantly medical in focus. Only 28 out of 225 content units (12%) from the clinician book discussed personal topics and only 3 out of 150 content units (2%) from the ask-the-doctor message board did so (see Table 4). Although patient sources were more variable, the minimum proportion of personal topics discussed in the patient sources (13/50 content units, 26%, from patient message board C) was more than twice the maximum proportion of personal topics discussed in clinician sources (28/225 content units, 12%, from the clinician book).

The most common medical topic discussed across all sources was “understanding biomedical concepts and processes,” making up 49 out of 150 content units (33%) discussed in patient message boards and 6 out of 210 content units (3%) in patient books, as well as 102 out of 150 content units (68%) discussed in the ask-the-doctor message board and 145 out of 225 content units (65%) in the clinician book (see Table 3). Within this medical subtopic, correspondents on patient message boards discussed clinical procedures and side effects, test results, and research or news articles about breast cancer. For example, after describing their medical history one correspondent asked “what is DIEP reconstruction?” Another correspondent asked whether “routine bone scans” are a standard part of long-term follow-up for breast cancer metastasis. Another correspondent looked for other patients in a similar situation to double-check what her “next step should be” in treatment. Many correspondents, particularly from message boards B and C, discussed research or news articles about various breast cancer treatments. In contrast, discussion on the ask-the-doctor message board revolved mostly around understanding the diagnosis and prognosis of breast cancer. For example, after describing aspects of their medical histories, correspondents commonly asked whether symptoms they were experiencing, such as “burning,” “aching,” or “swollen” breasts, could signal breast cancer.

The most common personal topic discussed across all sources was “managing personal tasks and projects,” making up 16 out of 150 content units (11%) among patient message boards and 86 out of 210 content units (41%) in patient books, as well as 1 out of 150 content units (<1%) in the ask-the-doctor message board and 19 out of 225 content units (9%) in the clinician book (see Table 3). All but 1 content unit from clinician sources came from the clinician book and covered issues ranging from exercise, meditation, and diet to prostheses. The 1 content unit from the ask-the-doctor message board that discussed this personal topic pertained to dietary advice to “boost” blood counts. Content units from the patient message boards commonly pertained to self-care, such as managing hair loss and seeking a “good wig shop.” In contrast, the patient books focused more on gathering and organizing information to play an active role in health care and self-care activities, such as nutrition and poetry writing.

### Differences in Recommendations Offered by Sources of Expertise

Although content units from both patient and clinician sources offered recommendations falling under all 4 types (action strategies, knowledge, perspectives, and information resources), the proportions of those types differed significantly between patient and clinician sources (χ^2^_3_[N = 7209] = 482.1, *P* < .001). On average, patient sources offered a greater proportion of action strategies and perspectives but a smaller proportion of knowledge than clinician sources. However, both types of sources offered similar proportions of information resources on average. For instance, the proportion of perspectives from patient sources ranged from 46 out of 1365 recommendations (3%) in patient book 2 to 86 out of 555 of recommendations (15%) in message board A. In contrast, a maximum of only 79 out of 3573 recommendations (2%) from the clinician book offered perspectives (see Table 4). Furthermore, the maximum proportion of knowledge from patient sources was 368 out of 838 recommendations (44%) from patient book 1, whereas the minimum proportion of knowledge from clinician sources was 1753 out of 3573 recommendations (49%) from the clinician book (see Table 4).

When we compared message boards alone and books alone, we also found significant differences in the types of recommendations offered between patient message boards and the ask-the-doctor message board (χ^2^_3_[N = 1435] = 153.5, *P* < .001), as well as between the patient and clinician books (χ^2^_3_[N = 5774] = 274.9, *P* < .001). Table 3indicates that the patient message boards offered larger proportions of action strategies, perspectives, and information resources than the ask-the-doctor message board. Similarly, the patient books offered larger proportions of action strategies, perspectives, and information resources than the clinician book.

When we delved further into the styles used to express recommendations, we found that action strategies, knowledge, and perspectives were frequently expressed implicitly through personal narratives in sources of patient expertise compared with the prescriptive style that was common to sources of clinician expertise. This difference in style was significant for action strategies (χ^2^_1_[N = 1589] = 281.4, *P* < .001), knowledge (χ^2^_1_[N = 3024] = 621.0, *P* < .001), and perspectives (χ^2^_1_[N = 390] = 49.1, *P* < .001). Nearly half of all action strategies from patient sources were narrative in style, whereas almost all action strategies from clinician sources were prescriptive in style (see Table 3). The same pattern holds between sources for recommended knowledge and recommended perspectives.

Although sources of patient expertise and sources of clinician expertise offered similar proportions of information resources on average, the types of information resources that were most commonly exchanged differed between those sources. For example, sources of patient expertise offered more webpages, poems, quotes, and news articles, but fewer books, contact information, and academic journals or research articles than sources of clinician expertise (see Table 3).

## Discussion

Results from this analysis show that patient expertise differs significantly from clinician expertise in topic (medical, personal, or both), type of recommendation (action strategies, knowledge, perspectives, and information resources), and style of recommendation (narrative vs prescriptive). Sources of clinician expertise were predominately medical in topic, knowledge-oriented in type, and prescriptive in style, whereas sources of patient expertise contained more personal topics that were carried through narrative-style action strategies and perspectives. These findings suggest that patients, by sharing their expertise about personal health, meet an important information need unmet by clinician sources. Our findings extend prior analyses of patient interaction with supportive evidence that differentiates patients’ experiential knowledge about personal health from the medical expertise of professionals. This contribution enhances our understanding about the fundamental nature of patient expertise and guides the design of peer-support tools that facilitate patient-expertise sharing.

**The Unique Nature of Patient Expertise**

Differences in the characteristics of patient expertise and clinician expertise support the notion that patients and health professionals possess different domains of health expertise [51]. Rather than filling the role of an amateur doctor (ie, claiming professional-like medical knowledge about the treatment of disease without having professional training), the experiential knowledge offered by patients appears to focus on coping with highly personal issues drawn from the context of daily life. This characterization of expertise over managing the personal side of health supports the claim that such knowledge is gained not through professional training, but rather through the trial and error of managing the lived experience of illness [1,3]. Reports on the expertise of patient groups who manage conditions other than cancer, such as epilepsy [24], share a similar illustration of the strength of expertise on the personal side of health. Although some patient expertise appears condition specific (eg, tips for managing hair loss from chemotherapy), other expertise appears transferable between patient groups (eg, what to look for in a clinician).

In addition to clinician expertise obtained from health professionals, patients are finding new ways to reach out to other patients to exchange complementary personal health advice based on their own experiences through collaborative tools on the Internet [27-31]. Our findings suggest that patients are filling an important and valuable function that is not fully served by traditional health care and medical information delivery models that lack focus on the personal side of health. Thus, patients could benefit from informatics tools designed to help them share their expertise with one another. Our findings provide a strong foundation for designing new patient-centered tools that meet patients’ needs for sharing expertise with peers.

### Design Implications for Patient Expertise-Sharing Tools

Patient expertise-sharing tools are technologies that bring patients together to interact and exchange their personal health knowledge. Enhancements to tools that patients already use to exchange personal health information, such as health-related social software [27-31], are sensible targets for facilitating patient-expertise sharing. Blogs, wikis, forums, social networking tools, and other collaborative tools are being increasingly used by patients to exchange personal health information [28,30,31]. For example, patients contribute and rate recommended websites on health-related wikis [52], exchange health-related information on Facebook [53-56], track and share their condition-specific symptoms and treatments through profiles with health-specific social networking tools [32], and search member directories to find patients who share the same diagnosis [57,58]. Given the high prevalence of seeking health advice from peers on the Internet [30,31], designers should explore enhancements that will make the expertise of patients more prominent, explicit, and accessible.

Design efforts to facilitate patient-expertise sharing can offer patients opportunities to interact with these collaborative technologies in ways that extend beyond the traditional, text-based message boards of the past. For example, participatory design work illustrates patients’ strong desire for online collaboration and networking tools, such as Facebook [56] or Myspace [59], to connect and share common illness experiences and valuable health resources [60,61]. Our findings on the fundamental nature of patient expertise provide a strong foundation for such innovative design efforts. In particular, as we detail in the following subsections, our findings have bearing on enhancing social software by including support for (1) collaboratively managing shared resources, (2) locating patient expertise, and (3) safeguarding against misinformation (Table 5).

**Table 5:** Design opportunities to facilitate patient-expertise sharing

Type of support	Design feature
Collaboratively managing shared resources	Common space to share and interact with varied resources
	User-generated tags and folksonomies that are meaningful to patients
	Methods for rating and recommending tailored resources
	Narrative and template formats for sharing experiences and expertise
Locating patient expertise	Detailed user profiles that illustrate areas of experience and expertise
	Methods for people finding and matchmaking
	Analytic tools for identifying topics of interest from user contributions
Safeguarding against misinformation	Features that preserve natural safeguarding strategies in a public context
	Change log to provide audit trail of corrections to content
	Vetting features to note affirmation, rebuttal, or reference sources

#### Collaboratively Managing Shared Resources

Designers should focus on developing common spaces for patients to manage the multitude of information resources they share together. The wide range of information resources (eg, webpages, books, articles, and multimedia) that patients exchange suggests the need for tools that enable patients to work together to create, annotate, store, share, and reuse content across a diverse range of formats and topics. Patients need help managing this full range of resources they recommend to and garner from one another. Collaborative features of social software, such as user-generated tags to organize content shared through a wiki, have the potential to support this need for collaboratively managing shared resources. For example, Weiss and Lorenzi [62] synthesized community wisdom about local cancer programs and services using collaborative Web-based tools for sharing community-based cancer resources. Others are developing recommendation systems for patients [63], such as a tag-based recommendation system that leverages community ratings of health content to rank tailored suggestions it provides to users [64].

Collaborative recommendation systems like these help users share their expertise by rating resources and benefit from each other’s views, opinions, and experiences through collaborative filtering [65]. We envision this collaborative space much like an updated version of an “expert patient knowledge base” [24]. Our findings on the breadth of information resources that patients exchange (eg, books, contacts, news and academic articles, poems and quotes, and recipes) suggest that incorporating collaborative recommendation methods into health-related social software could help users work together to manage this range of content and recommend useful resources to one another.

Given the range of medical and personal topics discussed among patients in our analysis, medically oriented resources (eg, medical dictionaries and patient information summaries) would certainly make up a valuable component of collaboratively managed collections of patient resources. However, the prominence of personal topics (eg, tracking medical expenses, working during treatment, what to tell your children, and selecting wigs) suggests that a fundamental component of such collections must include nonclinical resources as well. These resources should provide advice on personal topics related to work, family, the home, and social relationships in the context of illness. For example, one of the threads we analyzed consisted of dozens of suggestions from patients on considerations to make when writing an “end of life memoir” for family members (eg, your favorite books, family heirlooms, hobbies you enjoy, and world travels). Other examples include discussions about favorite “juicer recipes” and “experiences with sick-leave policies.” The breadth of these personal topics could link to a full range of relevant information resources from multiple domains (eg, medicine, law, social work, art, cooking, community resources, and finance). Users could tag and annotate these resources collaboratively in ways that capture important contextual ties to their specific experiences and facilitate later reuse by other users [66]. 

A medical library model [67] might provide only a partial fit for organizing the breadth of information needs met by the collections of resources shared among patients [68]. Our findings expand on work that shows a poor mapping between many concepts that patients use and controlled medical terminologies, such as the Unified Medical Language System (UMLS) metathesaurus [69]. For example, UMLS was not designed to capture many of the nonmedical concepts, such as the family, work, and social matters, for which patients turn to one another for help. Alternative organizational structures could allow users to compile shared information resources in personally meaningful, yet diverse, ways. For example, tools could encourage users to create *consumer health folksonomies* [70] to organize documents around their own conceptualization of health-related issues. Such folksonomies might resemble collaboratively constructed and tag-based systems that have emerged in other contexts, such as Web bookmarking [71].

Our findings provide additional insights for supporting collaboratively managed collections of resources. The common style of personal stories used to express patient expertise (see also [21,72]) suggests the potential value of narrative-based formats, such as “war stories,” that have been a highly valued format for sharing expertise in some professional work settings [73]. Repositories of personal health stories that are surfacing through social software, such as personal blogs [29] and CarePages’ “Stories of Inspiration” [74], might facilitate a natural expression of patient expertise and provide contextual detail upon which to create experience maps that guide patients’ problem solving surrounding specific health situations. Vetting features (ie, ratings, awards, and crowdsourcing techniques), whereby users associate comments or affirmations with personal stories, could help users to assess the fit of implicit advice those personal stories provide to their own health situations. Furthermore, patient expertise in the key form of action strategies could be exchanged through “how to” pages [75] that communicate strategies for dealing with personal health issues, or through templates that provide guidance by scaffolding action plans around personal health activities (eg, a preparing-for-surgery checklist). Patients could later recommend useful personal health practices through such templates to other patients.

##### Locating Patient Expertise

Designers should focus on developing tools that help patients find and connect with other patients who have specific kinds of experiences or expertise. As health-related use of social software grows [28,30,31], patients will need help locating other patients with the specific expertise they seek. During our analysis, for example, we observed patients posting requests to find other users with specific experiences or wisdom (eg, has anyone on this forum dealt with this particular rare side effect?). A common complaint about message boards is the challenge of determining who knows what, because the expertise of users gets lost within the volumes of threads in the forum. Design enhancements that make users’ requests and their expertise more explicit [8,34] will help to overcome these limitations.

Features of social networking tools, such as user profiles, can help bring users’ expertise to the surface, enabling a targeted search for patients with specific health and personal characteristics [34]. For example, a closer match in lifestyle and belief system leads to peer support that is perceived as more helpful [76]. Yet, most user profiles of social software are limited to a single health condition and a small set of demographics, and provide little if any indication of the kinds of expertise users can offer. Whether a user finds other patients for advice by posting personal health data to their personal profile [32] or by posting forum questions, this common broadcast strategy works only if people with the requisite expertise notice and respond. Once the user garners that expertise, they must determine the suitability of peers who provided the expertise for meeting their specific needs. Although traditional user profiles and broadcast mechanisms help, findings from our analysis point to expertise cues that could facilitate locating patient expertise through people-finding or matchmaking features. For example, user profiles could display the topics a user commonly discusses or information resources (eg, webpages, articles, and books) that users recommend one another but otherwise get buried within threads [34]. Consider a message board thread we analyzed in which a patient provided an extensive critique on a recent article about access to breast cancer treatments. Given a user profile, this patient could post the recommended article and her critique, thus making them visible and easily accessible to other users later.

We could also leverage the solid foundation of expertise-sharing research conducted in other settings to make progress in this important design direction of supporting the locating of patient expertise. For example, when confronted with an unfamiliar problem, people in professional work settings locate needed expertise by identifying potential sources (eg, other people and artifacts) and selecting specific sources to approach for help [77]. This practice of locating expertise has informed the design of tools that help professionals find colleagues with the desired, and often specialized, knowledge within a professional organization through features, such as user profiles and social networks [78-80]. With guidance from related work on expertise locators and our understanding of the characteristics of patient expertise, we can enhance social software and make it easier for patients to identify other users who have the specific patient expertise they need. To this end, we envision matchmaking tools that could support the following scenario:

Sally seeks advice about whether to work during chemotherapy. She wants to locate a patient who has already dealt with this situation (eg, “I want to find another mother of school-aged children who worked throughout chemotherapy”). She enters age, gender, and condition into a directory search service offered by a social networking tool for cancer patients. Unfortunately, she is overwhelmed by the large number of user profiles the system returns, which she must now manually review to find another user with the specific characteristics she is looking for. In particular, Sally needs awareness of not only the health condition and demographics of other users, but details about their specific knowledge and health-related experiences to answer questions, such as “Does this person have the experience I am interested in? If so, how recently? What is their experience level?”

Enhanced features that make specific and detailed health experiences of users more prominent could make Sally’s work much easier and tailored to her needs. For example, it was common for correspondents in the message boards we analyzed to preface their thread postings with detailed descriptions of their health experiences (eg, “I’m a 4-year survivor...”). Such details could be combined with a larger range of medical and nonmedical characteristics [32] to extend user profiles. Our findings also reveal a range of personal topics that patient expertise reflects (eg, managing health-related issues that connect to work or the home). We could also facilitate patient-expertise sharing by incorporating users’ topical expertise, based on the topics they discuss, into their user profiles. Access to both topical expertise and specific health experiences of other users provides important contextual cues that patients need to locate expertise that meets their specific needs [76,81].

#### Safeguarding Against Misinformation

Designers should also focus on features that preserve and encourage self-correction, self-monitoring, and other natural safeguarding strategies already used by patients online. Some might fear that enhancing informatics support for patient-expertise sharing could lead to the spread of mistaken, misinterpreted, outdated, incomplete, or otherwise poor-quality information. Although the potential for medical misinformation certainly exists, studies have examined patient interactions in online health communities and found minimal levels of medical misinformation [24,82-85].

We did not assess the accuracy of information exchanged in the patient message boards that we analyzed, but our observations of message board correspondents were consistent with previous research on strategies used to actively safeguard against the potential for misinformation, such as self-correction [83,86] and warnings from watchful members [23]. We also observed correspondents using additional safeguarding strategies, including source referencing (eg, “my oncologist told me that...”), advice prefacing (eg, “everyone has a different experience, [but this is what happened to me]”), rebuttal (eg, “our support group has many women’s experiences that prove otherwise”), and affirmative vetting of advice offered by other correspondents (eg, “I agree with all the ladies so far”).

Our observations point to the importance of preserving functionality that encourages patients’ natural misinformation-safeguarding strategies, such as vetting features within a public context, as health-related social software evolves to support patient-expertise sharing. In particular, our observations suggest support for audit trails that make content changes explicit and easy to log, and simple vetting features for noting affirmation or rebuttal (eg, thumps up/down) and for referencing source material.

### Study Limitations and Directions for Future Research

The characteristics of patient expertise we present are derived from a deep exploration of content from selected message boards and books in the context of cancer. The codebook resulting from our analysis is necessarily shaped by diverse interests and viewpoints of book authors and message board correspondents from the sources we analyzed. For example, expertise captured from a book written by a single author might not be as diverse or transferable as the expertise of multiple voices from a book coauthored by several people. Thus, our findings might not capture the breadth of expertise across the wide array of resources available to patients. For example, message boards reflect patients’ information needs through discussion that is initiated by patients’ own questions or offers of support. In comparison, books written by cancer survivors could provide a less direct reflection of authors’ and publishers’ perceptions about patients’ needs. Furthermore, individual content sources vary in their predominance of personal topics. Despite these differences, we found a strikingly similar distinction between the patient expertise in both books and message boards and the expertise in clinician sources. While our findings illustrate the volume of patient discussion beyond the medical realm of personal health, additional research is needed to tease out issues of misinformation and deeper distinctions within medical topics discussed.

Although our in-depth effort was scoped to small diverse samples, the work yielded rich descriptions that provide a solid basis for understanding patient expertise as a critical facet within the breadth of patients’ information needs. Details of our codebook point to a range of information needs related to the personal side of health and contribute to a holistic view of the patient. Given the experiential nature of patient expertise, it is plausible that the characteristics we ascribe to this specialized form of knowledge are also reflected by the experiential knowledge that people develop from personally managing other health situations, such as diabetes, heart disease, or pregnancy. Although aspects of patient expertise we identified are specific to cancer, other aspects could be widespread and shared by patients with other conditions [24]. Future research could explore which characteristics of patient expertise reflected by our codebook are transferrable to these other health contexts.

Future work could also explore how our design implications play out within patients’ expanding space of social participation on the Internet [87]. Although our content analysis captured only a sampling of content sources, our enhanced understanding of patient expertise points to innovative directions for the design of peer-support tools that facilitate patient-expertise sharing. Our findings also provide insights for the design of tools that encourage information sharing between patients and health professionals, such as integrating a broad range of personal factors with health care planning in the context of shared decision making [10,11], as well as for the design of tools that bridge the expertise of patients and professionals [52]. 

### Conclusion

Our findings demonstrate that patient expertise differs significantly from the expertise of clinicians in topic, type, and style. Neither increasing the amount of time that patients spend with health care providers nor training patients with medical knowledge to become amateur doctors appears sufficient to meet the needs for patient expertise. Instead, we offer alternatives in the form of design directions for facilitating patient-expertise sharing with health-related social software. Patients provide other patients with a unique and valued information resource that complements expertise provided by health professionals. Patients deserve informatics support that can fill the breadth of their health information needs by facilitating this patient-expertise sharing.

## Abbreviations

UMLS: Unified Medical Language System
